# Dysregulated circulating microRNA‐126 in chronic obstructive pulmonary disease: linkage with acute exacerbation risk, severity degree, and inflammatory cytokines

**DOI:** 10.1002/jcla.24204

**Published:** 2022-01-21

**Authors:** Congying Wang, Dong Feng, Shanfeng Dong, Ruilian He, Bosheng Fan

**Affiliations:** ^1^ Department of Respiratory and Critical Care Medicine Jiaozuo Coal Industry (Group) Co. Ltd. Central Hospital Jiaozuo China; ^2^ Department of Orthopedics Jiaozuo Coal Industry (Group) Co. Ltd. Central Hospital Jiaozuo China; ^3^ Department of Urology Jiaozuo Coal Industry (Group) Co. Ltd. Central Hospital Jiaozuo China; ^4^ Department of Neurology Jiaozuo Coal Industry (Group) Co. Ltd. Central Hospital Jiaozuo China

**Keywords:** acute exacerbation, chronic obstructive pulmonary disease, GOLD stage, inflammatory cytokines, microRNA‐126

## Abstract

**Background:**

MicroRNA‐126 (miR‐126) is engaged in respiratory diseases via regulating airway tissue injury and pulmonary inflammation, while its relation with chronic obstructive pulmonary disease (COPD) is not reported. The study aimed to evaluate the value of miR‐126 for estimating COPD acute exacerbation risk and its relation to disease severity and inflammatory cytokines in COPD patients.

**Methods:**

This study was a case–control study. Seventy acute exacerbation COPD (AECOPD) patients, 70 stable COPD (SCOPD) patients, and 70 healthy controls (HCs) were consecutively recruited. Plasma miR‐126 expression was detected by reverse transcription quantitative polymerase chain reaction. Plasma tumor necrosis factor α (TNF‐α), interleukin‐1β (IL‐1β), interleukin‐6 (IL‐6), and interleukin‐17 (IL‐17) in COPD patients were further determined by enzyme‐linked immunosorbent assay.

**Results:**

MiR‐126 was higher in AECOPD patients compared to SCOPD patients and HCs (both *P*_adj_ < 0.001). Receiver operating characteristic curves revealed miR‐126 distinguished AECOPD patients from SCOPD patients (area under curve (AUC): 0.805, 95%CI: 0.733–0.877) and HCs (AUC: 0.884, 95%CI: 0.829–0.939) and also distinguished SCOPD from HCs (AUC = 0.656, 95%CI: 0.566–0.747). MiR‐126 positively related to GOLD stage in both AECOPD patients (*p *< 0.001) and SCOPD patients (*p *< 0.001). Furthermore, miR‐126 positively linked with TNF‐α (*p *< 0.001), IL‐1β (*p *= 0.002), IL‐6 (*p *= 0.009), and IL‐17 (*p *< 0.001) levels in AECOPD patients; but miR‐126 only positively related to TNF‐α and IL‐17 levels (all *p *< 0.050), instead of IL‐1β or IL‐6 level (all *p *> 0.050) in SCOPD patients and HCs.

**Conclusion:**

Dysregulated circulating miR‐126 not only relates to COPD susceptibility and its acute exacerbation risk but also links with disease severity and inflammatory cytokines in COPD patients.

## Graphical abstract

70 acute exacerbation COPD (AECOPD) patients, 70 stable COPD (SCOPD) patients and 70 health controls (HCs) were consecutively recruited. Their plasma miR‐126 and inflammatory cytokine levels were detected by RT‐qPCR and ELISA, respectively. MiR‐126 was higher in AECOPD patients compared to SCOPD patients and HCs (both *p* < 0.001). Receiver operating characteristic curves revealed miR‐126 distinguished AECOPD patients from HCs (AUC: 0.884) and SCOPD patients (AUC: 0.805), which also distinguished SCOPD from HCs (AUC = 0.656). MiR‐126 positively related to GOLD stage in AECOPD patients and SCOPD patients (both *p* < 0.001). Furthermore, miR‐126 positively linked with TNF‐α (*p* < 0.001), IL‐1β (*p* = 0.002), IL‐6 (*p* = 0.009) and IL‐17 (*p* < 0.001) levels in AECOPD patients; but the linkage was weaker in SCOPD patients and HCs. Conclusively, miR‐126 shows potency as biomarker for COPD.

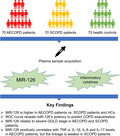

## 1 INTRODUCTION

Chronic obstructive pulmonary disease (COPD) is a chronic airway inflammatory disease mediated by different inflammatory cells, cytokines, or other mediators, with its main characteristics as the persistence of airway inflammation, destruction of lung tissue structure, airway remodeling, and decline of lung function.^1^, ^2^, ^3^ According to the recent report of the World Health Organization, there are about 251 million new COPD cases every year, and the incidence is still gradually increasing due to the increase of smoking and air pollution.^4^ Despite encouraging advances in early diagnosis and disease management in recent years, COPD remains the fourth leading cause of death worldwide.^1^, ^2^ Therefore, it is necessary to make efforts to explore effective markers to predict COPD risk and realize disease monitor, so as to improve the treatment outcome and quality of life in these patients.

MicroRNA (miRNA/miR) is a small RNA molecule with a length of about 22 nucleotides that binds to the 3 ‘untranslated region of target mRNA and then silences the transcribed gene expression, which participates in the process of immune and inflammatory responses.^5^, ^6^ As one of the most studied miRNAs with impressive regulatory functions, miR‐126 plays a key role in regulating endothelial cell function and angiogenesis, maintaining vascular integrity, as well as modifying pulmonary inflammation,^7^, ^8^, ^9^, ^10^, ^11^ which involved in the occurrence and progression of a variety of respiratory diseases, such as asthma, pulmonary hypertension, and pulmonary infections,^10^, ^12^, ^13^ but there are few reports on miR‐126 in COPD patients.

Therefore, this study aimed to evaluate the value of miR‐126 for estimating COPD acute exacerbation risk and its relation to disease severity and inflammatory cytokines in COPD patients.

## 2 METHODS

### 2.1 Participants

This study was a case–control study. This study consecutively enrolled 70 acute exacerbation COPD (AECOPD) patients, 70 stable COPD (SCOPD) patients, and 70 healthy controls (HCs) between January 2019 and March 2021. This study had been approved by the Ethics Review Committee, with signed informed consents provided by all subjects.

The inclusion criteria of AECOPD patients were (1) diagnosed as COPD according to GOLD criteria^14^; (2) classified as acute exacerbation phase according to “Expert consensus on acute exacerbation of chronic obstructive pulmonary disease in the People's Republic of China”^15^; and (3) age above 18 years. The inclusion criteria of SCOPD patients were (1) diagnosed as COPD according to GOLD criteria^14^; (2) classified as stable phase according to “Expert consensus on acute exacerbation of chronic obstructive pulmonary disease in the People's Republic of China”^15^; and (3) age above 18 years. All AECOPD patients and SCOPD patients with the following conditions were excluded: (1) complicated with serious infection; (2) suffering from solid tumors, hematological malignancies, or autoimmune diseases; and (3) pregnant or lactating women.

The inclusion criteria of HCs were: (1) physical examination results showed no obvious abnormalities in all physical examination indicators; (2) at least 18 years of age; (3) without history of severe lung infections/diseases, solid tumors, hematological malignancies, or autoimmune diseases; and (4) not pregnant or lactating women.

### 2.2 Data processing

After enrollment, basic characteristics including age, sex, body mass index (BMI), family history of COPD, and smoking history were recorded, as well as lung function test data that included expiratory volume in 1 s/forced vital capacity (FEV_1_/FVC) and FEV_1_ (%pred). In addition, according to FEV_1_ (%pred) data, COPD patients were classified as GOLD stage 1 (FEV_1_ (%pred) ≥80%), stage 2 (50%≤FEV_1_ (%pred) <80%), stage 3 (30%≤FEV_1_ (%pred) <50%), and stage 4 (FEV_1_ (%pred) <30%).

### 2.3 Samples and detections

Peripheral blood samples were collected from each subject after being enrolled in this study. Then, the plasma was separated within 1 h after the collection of peripheral blood samples; subsequently, all samples were stored at −80℃ for no more than 1 year. After that, at the end of each year, all samples were defrosted from the −80℃ to propose to reverse transcription quantitative polymerase chain reaction (RT‐qPCR) for miR‐126 detection and cytokines measurement.

Total RNA was extracted from plasma using the QIAamp RNA Blood Mini kit (Qiagen, Duesseldorf, Germany) and then reversely transcribed into cDNA via the PrimeScript™ RT Master Mix kit (Takara, Dalian, China), and finally PCR was conducted using a TB Green™ Fast qPCR Mix kit (Takara, Dalian, China). The reaction conditions were pre‐denaturation at 95℃ for 5 min, denaturation at 95℃ for 5 s, annealing at 61℃ for 15 s, extension at 72℃ for 30 s, and 40 cycles. Using U6 as internal reference, the relative expression of miR‐126 was calculated by 2^−△△Ct^ method. Primer information was as follows: miR‐126, forward primer: ACACTCCAGCTGGGCATTATTACTTTTGGTAC, reverse primer: ACACTCCAGCTGGGACTGCAGTGAAGGCACTT; U6, forward primer: CTCGCTTCGGCAGCACA, reverse primer: AACGCTTCACGAATTTGCGT.

Tumor necrosis factor α (TNF‐α), interleukin‐1β (IL‐1β), interleukin‐6 (IL‐6), and interleukin‐17 (IL‐17) were detected using commercial enzyme‐linked immunosorbent assay kits (Abcam, Massachusetts, USA) according to their instructions.

### 2.4 Statistics

Data were described as mean and standard deviation, median value, and inter‐quartile range (IQR) or frequency (percentage). Comparisons between/among groups were performed using one‐way ANOVA, Chi‐square test, Wilcoxon rank sum test, or Kruskal–Wallis test. Spearman rank correlation coefficient test was used for correlation analysis. The efficiency of miR‐126 in distinguishing different subject was analyzed by receiver operating characteristic curve with AUC. All tests were two‐sided, and *p *< 0.05 indicated statistical significance.

## 3 RESULTS

### 3.1 Patients’ characteristics

AECOPD patients exhibited the age of 67.8 ± 6.3 years, with 53 males/17 females; SCOPD patients showed the age of 67.4 ± 7.9 years, with 51 male/19 females; HCs revealed the age of 68.0 ± 7.0 years, with 47 male/23 females. By three‐group comparison, age (*p *= 0.887), gender (*p *= 0.517), and BMI (*p *= 0.183) were of no difference among them (Table 1). More detailed information about lung function and inflammatory cytokines is presented in Table 1.

**TABLE 1:** Clinical characteristics

Parameters	AECOPD patients (*n* = 70)	SCOPD patients (*n* = 70)	HCs (*n* = 70)	*P* value
Age (years), mean ±SD	67.8 ± 6.3	67.4 ± 7.9	68.0 ± 7.0	0.887
Gender (male/female), No.	53/17	51/19	47/23	0.517
BMI (kg/m^2^), mean ±SD	23.7 ± 3.1	22.0 ± 2.9	22.8 ± 2.6	0.183
History of COPD, no. (%)	23 (32.9)	24 (34.3)	8 (11.4)	0.003
Smoke, no. (%)	37 (52.9)	32 (45.7)	20 (28.6)	0.011
FEV_1_/FVC (%), median (IQR)	59.6 (53.7–65.6)	61.6 (57.5–65.2)	82.3 (79.6–84.3)	<0.001
FEV_1_ (%pred), median (IQR)	55.4 (46.9–77.1)	66.2 (56.0–82.4)	99.0 (96.3–100.8)	<0.001
GOLD stage, no. (%)				0.161
Stage 1	17 (24.3)	27 (38.6)	–	
Stage 2	34 (48.6)	30 (42.9)	–	
Stage 3	19 (27.1)	13 (18.6)	–	
TNF‐α (pg/ml), median (IQR)	61.5 (36.0–96.9)	19.4 (9.8–35.3)	13.0 (8.4–20.1)	<0.001
IL−1β (pg/ml), median (IQR)	4.1 (2.2–6.7)	1.3 (0.6–2.2)	0.9 (0.5–1.3)	<0.001
IL−6 (pg/ml), median (IQR)	39.2 (16.2–59.4)	9.2 (4.9–19.0)	6.4 (3.9–10.5)	<0.001
IL−17 (pg/ml), median (IQR)	52.0 (22.1–127.1)	20.8 (6.2–35.0)	9.7 (5.9–16.6)	<0.001

### 3.2 Dysregulated miR‐126 in COPD patients

**FIGURE 1:**
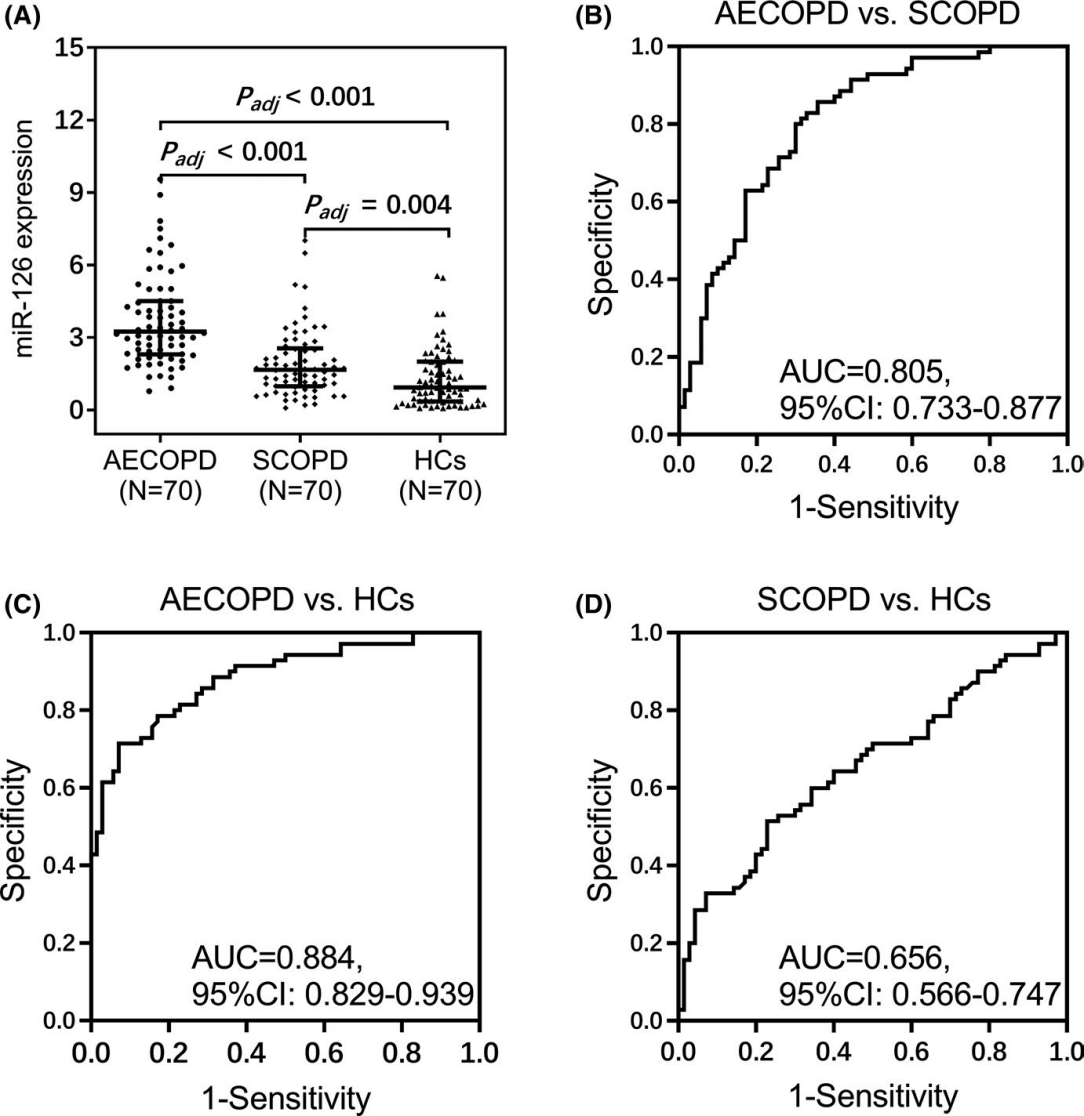
MiR‐126 expression among acute exacerbation chronic obstructive pulmonary disease (AECOPD) patients, stable COPD (SCOPD) patients, health controls (HCs). Comparison of plasma miR‐126 expression among AECOPD patients, SCOPD patients, and HCs (A). Value of miR‐126 expression in distinguishing AECOPD patients from SCOPD patients (B), AECOPD patients from HCs (C), and SCOPD patients from HCs (D)

MiR‐126 was increased in AECOPD patients compared to HCs (*P*_adj_ < 0.001) and SCOPD patients (*P*_adj_ < 0.001) and also elevated in SCOPD compared with HCs (*P*_adj_ = 0.004) (Figure 1A). Besides, miR‐126 showed the potential to distinguish AECOPD patients from SCOPD patients with AUC 0.805 (95%CI: 0.733–0.877), AECOPD patients from HCs with AUC 0.884 (95%CI: 0.829–0.939), and SCOPD patients from HCs with AUC 0.656 (95%CI: 0.566–0.747) (Figure 1B‐D). Further univariate logistic regression analysis showed that miR‐126 was associated with elevated COPD risk (odds ratio: 2.088, *p *< 0.001, Table S1).

### 3.3 MiR‐126 related to GOLD stage in COPD patients

AECOPD patients with GOLD stage 1, stage 2, and stage 3 presented with miR‐126 expression of 2.030 (IQR: 1.718–3.330), 3.230 (IQR: 2.325–4.144), and 5.205 (IQR: 2.965–7.107), and then correlation analysis revealed miR‐126 positively correlated with GOLD stage in AECOPD patients (*p *< 0.001) (Table 2). Besides, further analysis also disclosed that miR‐126 positively related to GOLD stage in SCOPD patients (*p *< 0.001).

**TABLE 2:** Correlation between miR‐126 and GOLD stage

Parameter	miR−126, median (IQR)	
	AECOPD patients	SCOPD patients
GOLD stage		
Stage 1	2.030 (1.718–3.330)	0.948 (0.564–1.271)
Stage 2	3.230 (2.325–4.144)	1.852 (1.423–2.655)
Stage 3	5.205 (2.965–7.107)	2.353 (1.667–4.270)
*P* value	<0.001	<0.001

### 3.4 MiR‐126 linked with inflammatory cytokines in COPD patients

MiR‐126 positively linked with TNF‐α (*p *< 0.001), IL‐1β (*p *= 0.002), IL‐6 (*p *= 0.009), and IL‐17 (*p *< 0.001) levels in AECOPD patients (Table 3). But miR‐126 only positively related to TNF‐α and IL‐17 levels (all *p *< 0.050), instead of IL‐1β or IL‐6 level (all *p *> 0.050) in SCOPD patients and HCs.

**TABLE 3:** Correlation between miR‐126 and inflammatory cytokines

Cytokine	miR−126					
	AECOPD patients		SCOPD patients		HCs	
	*P* value	r	*P* value	r	*P* value	r
TNF‐α	<0.001	0.430	0.016	0.287	0.014	0.292
IL−1β	0.002	0.356	0.227	0.146	0.063	0.223
IL−6	0.009	0.310	0.090	0.204	0.050	0.235
IL−17	<0.001	0.406	0.006	0.327	0.006	0.328

## 4 DISCUSSION

MiRNA is a group of small non‐coding RNA molecules, which can directly participate in the regulation of various physiological and pathological processes such as cell proliferation, differentiation, and apoptosis by inhibiting or degrading the mRNA translation of target genes.^16^ In recent years, studies have found that the maladjustment of a variety of miRNAs can lead to dysplasia of lung tissue and decline of immune function, which is closely related to the etiology and progression of various lung diseases, especially COPD.^17^, ^18^ For example, miR‐203 catalyzes subunit through TGF‐activated kinase (TAK1) and phosphatidylinositol‐3‐kinase (PI3KCA) to inhibit nuclear factor κB, resulting in the occurrence of COPD.^19^ In addition, miR‐3202 inhibits apoptosis of human bronchial epithelial cells and reduces interferon‐γ by inhibiting Fas apoptotic inhibitory molecule 2 and TNF‐α levels.^20^ What is more, miR‐190a‐5p is involved in the occurrence of hypoxia‐induced pulmonary hypertension by upregulating Kruppel‐like factor 15, thus aggravating COPD with pulmonary hypertension.^21^ These studies indicate the essential roles of miRNAs engaged in COPD.

MiR‐126, as an endothelium‐specific miRNA, can directly bind to DNA and prevent mRNA transcription, translation, and degradation.^22^, ^23^ Despite that a large number of studies have elucidated the role of miR‐126 in various cancers, inspiring progress has also been made about the involvement of miR‐126 in respiratory diseases in recent years.^12^, ^24^, ^25^ For example, it is observed that the expression of miR‐126 is obviously increased in the airway of mouse asthma models sensitized by house dust mite (HDM); considering that the main sensitizing component of HDM is toll‐like receptor 4 (TLR4) signaling pathway that activates lipolysaccharides in the immune inflammatory response, the TLR4 is then knocked out, which disclosed that no significant change in the expression of miR‐126 in HDM sensitized mice, suggesting that miR‐126 may be involved in the pathological process of asthma through upregulation of TLR4.^12^ Another study discovers that miR‐126 deficiency upregulates sprouty‐related EVH1 domain‐containing protein 1 (SPRED‐1) to reduce the activation of RAF kinase and mitogen‐activated protein kinase, thereby inhibits the vascular endothelial growth factor pathway, and subsequently aggravates pulmonary hypertension with right ventricular failure.^26^ Furthermore, several studies have found that miR‐126 also plays a pro‐inflammatory role in respiratory diseases. For instance, miR‐126 expression in the airways of ovalbumin sensitized mice is increased and remained unchanged for 4 weeks, suggesting that the high expression of miR‐126 plays a crucial role in airway inflammation in bronchial asthma.^24^ Of note, miR‐126 has been found to release IL‐13 by activating T cells, thereby exacerbating inflammation and developing many of the characteristics of asthma (such as airway hyperresponsiveness, hypersecretion of mucus, airway eosinophilic, and B cell activation).^27^, ^28^

Several studies reveal that aberrant expression of miR‐126 in patients with respiratory diseases shows potency to be diagnostic biomarker, such as bronchial asthma and non‐small cell lung cancer.^24^, ^25^ Considering that miR‐126 may be directly involved in airway pathology and its pro‐inflammatory role in a variety of respiratory diseases, we speculated that miR‐126 may relate to the development of COPD,^12^, ^24^, ^25^, ^26^, ^27^, ^28^ but its studies in COPD patients have rarely been reported. Therefore, this study detected the expression of miR‐126 in COPD patients and evaluated its predictive value for COPD acute exacerbation. We found that the expression of miR‐126 in AECOPD patients was higher than that in SCOPD patients and HCs, which also disclosed a good predictive value for the risk of COPD, as well as its acute exacerbation. This may be due to the fact that miR‐126 can promote the release of inflammatory cytokines by regulating multiple genes (such as IL‐13) and signaling pathways (such as TLR4 signaling pathway), thus aggravating the inflammatory response and further increasing the risk of COPD and its acute exacerbation.^12^, ^27^, ^28^

A few studies also report the correlation between miR‐126 expression and disease severity in patients with other respiratory illness but not COPD. For example, in patients with bronchial asthma, high expression of miR‐126 associates with FEV_1_/FVC.^24^ Similar to the above results, we found that miR‐126 expression was positively correlated with GOLD stage in AECOPD patients and SCOPD patients. This may result from: (1) miR‐126 directly affects airway pathology through regulating of several genes (such as SPRED‐1) and signaling pathways (such as TLR4 signaling pathway), thus aggravating the disease severity of COPD patients^12^, ^26^; (2) miR‐126 promotes the release of inflammatory factors, which leads to acute and chronic airway inflammation, and subsequently causes pathophysiological changes such as reversible airway restriction, airway remodeling, and airway hyperresponsiveness, then exacerbates disease severity in COPD patients.^27^, ^28^ These also explain the subsequent findings that miR‐126 related to inflammatory cytokines in COPD patients especially AECOPD patients in our study.

There were still some limitations in this study: (1) 70 AECOPD patients, 70 SCOPD patients, and 70 HCs were included in this study, whose sample size was small that might affect the statistical power of the analyses, besides it is hard to determine the correlation of miR‐126 with some of the complications of COPD such as pulmonary hypertension in COPD patients. (2) This study was a single‐center study that might exist patients’ selection bias and assessment bias, so further multiple‐center study was needed in the future. (3) The specific mechanism of miR‐126 in COPD pathogenesis was still unclear, and further study was necessary. (4) miR‐126 level in the sputum or bronchoalveolar fluid should be measured in our forthcoming study. (5) There were no follow‐up data in this study; thus, it was hard to determine the change of miR‐126 or its prognostic value in COPD which needed to be analyzed in further study. (6) The miR‐126 level in other respiratory diseases could also be determined in the forthcoming study.

In conclusion, dysregulated circulating miR‐126 not only relates to COPD susceptibility and its acute exacerbation risk but also links with disease severity and inflammatory cytokines in COPD patients indicating that the miR‐126 might serve as a potential biomarker for revealing the COPD risk and their disease severity. This study could help the clinicians to stratify the COPD patients and individualize the treatment for COPD patients.

## CONFLICTS OF INTEREST

The authors declare that they have no conflicts of interest.

## Supporting information

Table S1

## Data Availability

Data sharing not applicable to this article as no datasets were generated or analyzed during the current study.
