# Supplementary material for: Dysregulated circulating microRNA‐126 in chronic obstructive pulmonary disease: linkage with acute exacerbation risk, severity degree, and inflammatory cytokines
Source: J Clin Lab Anal. 2022 Jan 21;36(3):e24204. doi: 10.1002/jcla.24204 (PMC8906012; doi:10.1002/jcla.24204)
Supplement: Supplementary file 1 — Table S1 [file JCLA-36-e24204-s001.docx]

**Supplementary table 1.** Factors predicting the COPD risk by univariate logistic regression analysis.

| Items | *P* value | OR | 95% CI | |
| --- | --- | --- | --- | --- |
|  |  |  | Lower | Upper |
| miR-126 | <0.001 | 2.088 | 1.580 | 2.760 |
| Age (years) | 0.694 | 0.992 | 0.952 | 1.033 |
| Gender (male vs. female) | 0.279 | 1.414 | 0.756 | 2.645 |
| BMI (kg/m^2^) | 0.301 | 0.948 | 0.857 | 1.049 |
| Family history of COPD | 0.001 | 3.917 | 1.733 | 8.854 |
| Smoke | 0.005 | 2.430 | 1.313 | 4.496 |
| FEV1/FVC (%) | 0.982 | <0.001 | <0.001 | 3.170E+296 |
| FEV1 (pred) (%) | 0.962 | <0.001 | <0.001 | 1.394E+207 |
| TNF-α (pg/ml) | <0.001 | 1.085 | 1.054 | 1.118 |
| IL-1β (pg/ml) | <0.001 | 3.187 | 2.067 | 4.912 |
| IL-6 (pg/ml) | <0.001 | 1.103 | 1.059 | 1.149 |
| IL-17 (pg/ml) | <0.001 | 1.052 | 1.029 | 1.075 |
